# Correlation between clinical findings and results of autologous serum skin test in patients with chronic idiopathic urticaria

**DOI:** 10.1186/1710-1492-7-S2-A10

**Published:** 2011-11-14

**Authors:** Soheyla Alyasin, Ali Asghar Karimi, Ali Amiri, Mohammad Javad Ehsaei, Fariborz Ghaffarpasand

**Affiliations:** 1 Department of Pediatrics, Shiraz University of Medical Sciences, Shiraz, Iran; 2 Student Research Committee, Fasa University of Medical Sciences, Fasa, Iran

## Objectives

To investigate the correlation between the clinical characteristics of chronic idiopathic urticaria (CIU) and the results of autologous skin test (ASST).

## Methods

This was prospective observational study being performed in the Motahari allergy Outpatient Clinics, including 69 patients referring to these clinics with diagnosis of CIU. ASST was performed in all the patients. We compared the diseases characteristics including duration severity, number of urticaria, and size of urticaria between those with positive and negative results of ASST.

## Results

Of 69 patients with CIU 39 (56.5%) were females and 30 (43.5%) were males with mean age of 32.5 ± 12.3 (range 6–62) years. Patients with positive ASST had significantly higher numbers of wheals (*p*=*0.043*) with larger sizes (*p*=*0.031*). They also had higher frequencies of wheals (63.8% *vs.* 46.4% daily; *p*=*0.039*) and higher disease activity scores (1.57 ± 0.9 *vs.* 2.01 ± 1.6; *p*=*0.044*). The diseases activity score was positively correlated with size of the wheals (*r*=*0.561*, *p*=*0.002*), duration of the wheals (*r*=*0.436*, *p*=*0.013*), duration of the disease (*r*=*0.312*, *p*=*0.022*) and total number of wheals (*r*=*0.351*, *p*=*0.031*). Patients with positive ASST results had significantly higher frequencies of arthritis (*p*=*0.027*).

## Conclusion

Positive ASST results are associated with more severe disease determined by larger wheals' size, longer duration of the disease and the higher frequencies of the disease. As ASST reveals the autoimmune basis of the CIU, it can be concluded that CIU patients with autoimmune basis will suffer from more severe disease.

